# Expression profiles of hsa-miR-148a-3p and hsa-miR-125b-5p in human breast milk and infant formulae

**DOI:** 10.1186/s13006-021-00436-7

**Published:** 2022-01-03

**Authors:** Takeshi Chiba, Aya Kooka, Kiyoko Kowatari, Megumi Yoshizawa, Naoko Chiba, Akira Takaguri, Yoshiyuki Fukushi, Fuminori Hongo, Hideki Sato, Shinichiro Wada

**Affiliations:** 1 Faculty of Pharmaceutical Sciences, Hokkaido University of Science, 15-4-1, Maeda 7-jo, Teine-ku, Sapporo-shi, Hokkaido 006-8565 Japan; 2 Creation Research Institute of Life Science in KITA-no-DAICHI, Hokkaido University of Science, Sapporo-shi, Hokkaido Japan; 3 Department of Pharmacy, Teine Keijinkai Hospital, Sapporo-shi, Hokkaido Japan; 4 Department of Nursing, Teine Keijinkai Hospital, Sapporo-shi, Hokkaido Japan; 5 Ginza Pharmacy, Shibata-gun, Miyagi Japan; 6 Department of Obstetrics and Gynecology, Teine Keijinkai Hospital, Sapporo-shi, Hokkaido Japan

**Keywords:** Breast milk, microRNA, miR-148a, miR-125b, Infant formula, Lactation

## Abstract

**Background:**

Milk-derived microRNAs (miRNAs), including hsa-miR-148a-3p (miR-148a) and hsa-miR-125b-5p (miR-125b), have been shown to be beneficial to the gastrointestinal function in infants. Here, we investigated their expression during lactation in humans and determined whether the infant formulae available in Japan contain these miRNAs.

**Methods:**

Healthy Japanese women (*n* = 16) who gave birth vaginally or by cesarean section at the Teine Keijinkai Hospital between 1 September 2020, and 31 April 2021 were included in this study. Breast milk was collected by nurses on days 4 or 5 after delivery (hereinafter, transition milk) and on day 30 of postpartum (hereinafter, mature milk). The levels of miR-148a and miR-125b in breastmilk and six commercially available infant formulae were compared and evaluated using quantitative reverse transcription-polymerase chain reaction.

**Results:**

In all participants, the miR-148a level in mature breastmilk was significantly lower than that in the transition milk. The changes in miR-125b expression during lactation showed similar trends to the changes in miR-148a expression. The miR-148a and miR-125b levels in all analyzed infant formulae were lower than 1/500th and 1/100th of those in mature breastmilk, respectively.

**Conclusions:**

The levels of both miR-148a and miR-125b in human breast milk decreased on day 30 postpartum compared with those in the transition milk. Additionally, the expression of these miRNAs in infant formulae available in Japan was very low. Further studies with larger populations are required to understand precisely the lactational changes in the expression of miR148a and miR-125b in breast milk.

## Background

Breast milk is not only an indispensable source of nutrients for infant growth and development, but also contains various essential immunologic components with anti-infectious activities and critical roles in the formation of immunity [1]. Infants < 6 months who consume infant formulae exclusively, or in combination with breast milk (i.e., mixed milk), face higher risks of infectious morbidity and mortality during the first two years of life and are more susceptible to chronic diseases over the long term, when compared with infants in the same age group who consume only breast milk [2–5]. Therefore, various health organizations worldwide, including the United Nations Children’s Fund, World Health Organization, and the American Society of Pediatrics recommend that children should be exclusively breastfed for the first six months of life [6].

MicroRNAs (miRNA) are short (approximately 22 nucleotides long), non-coding RNAs found in various body fluids, such as the blood, saliva, urine, and milk. They control several biological processes in cells, including cell growth, differentiation, and apoptosis [7]. Additionally, miRNAs bind to target mRNAs and regulate gene expression by inhibiting translation [7].

Several hundred of miRNAs have been detected in breast milk, most of which are synthesized by mammary epithelial cells [8–11]. Milk exosomes enclose milk-derived miRNAs [12–14] and are capable of withstanding low pH and RNase treatment; this suggests that miRNAs enclosed by exosomes are protected against digestion in infant gastrointestinal tract. When fluorescence-labeled bovine milk-derived exosomes were orally administered to mice, the exosomes were detected in the brain, liver, lung, and spleen of the mice [15]. These observations show that milk exosomes and milk-derived miRNAs reach some tissues in infants via general circulation.

Among milk-derived miRNAs, hsa-miR-148a-3p (miR-148a) and hsa-miR-125b-5p (miR-125b) are abundant in human breast milk [11, 16]. These two miRNAs are considered beneficial to infants [11, 17]. A previous study showed that intragastric administration of porcine milk exosomes increased villus height and crypt depth in the intestine of mice [18], implying that milk exosomes contribute to gastrointestinal development in infants. Additionally, miR-148a expression in normal human colorectal cells (CRL-1831) treated with porcine milk exosomes significantly increased compared with that in control cells, and DNA methyltransferase 1 (DNMT1) expression in the colorectal cells significantly decreased compared with that in the control cells [9]. These findings imply that miR-148a is incorporated in milk exosomes and it decreased the expression of DNMT1, which is a direct target of miR-148a [11, 17]. Decreased expression of DNMT1 increased the expression of survivin, which inhibits the expression of apoptosis proteins [17]. Additionally, P53, which is a direct target of miR-125b and negatively regulates survivin expression via the induction of DNA methylation by DNMT1, was suppressed by miR-125b [19, 20]. These studies show that miR-148a and miR-125b in breast milk may contribute to the survival and proliferation of gastrointestinal cells in infants.

A recent study showed that miR-148a inhibited colitis and colitis-associated tumorigenesis via suppression of signaling of STAT3 and NF-κB, which are inflammation-associated factors [21]. Additionally, same study reported that miR-148a expression was downregulated in colon tissues in patients with inflammatory bowel disease [21]. Furthermore, miR-125b impaired the tight junction protein (ex. cingulin and claudin-2) whose expression is upregulated in jejunum in diarrhea-predominant inflammatory bowel syndrome [22]. These results imply that breast milk-derived miR-148a and miR-125b may be involved in the prevention of these bowel-associated diseases in infants.

A previous study reported that breast milk of mothers with a body mass index (BMI) > 25 kg/m^2^ had lower protein concentration than that of mothers with BMI < 25 kg/m^2^, and that the breast milk of mothers who smoke had significantly lower levels of proteins and lipids than that of mothers who do not smoke [23]. Furthermore, the levels of total nitrogen, protein nitrogen, sodium, chloride, and magnesium in the breast milk of mothers who had premature deliveries (< 37 weeks of gestational age) were significantly higher than those in the breast milk of mothers who had full term deliveries [24]. These findings suggest that smoking, obesity, and premature delivery influence the composition of breast milk.

To the best of our knowledge, the changes in miR-148a and miR-125b expression during lactation in humans have not been investigated to date. Additionally, whether these miRNAs are found in infant formulae commercially available in Japan has not yet been elucidated. Thus, in the present study, we evaluated and compared the expression of miR-148a and miR-125b in breast milk from healthy lactating women who did not have factors that could influence the composition of breast milk, on day 4 or 5 after delivery (hereinafter, termed transition milk), on day 30 after delivery (hereinafter, mature milk), and six commercially available infant formulae.

## Methods

### Study participants and milk sample collection

Healthy Japanese women aged between 20 and 40 years who had a vaginal delivery or a cesarean section at the Teine Keijinkai Hospital between 1 September 2020 and 31 April 2021, those who had no medication and supplements during pregnancy, those who did not have insufficient pituitary function, and those who were not obese (BMI < 25) before pregnancy, were included in this study. Additionally, women with premature delivery (< 37 weeks of gestational age) were excluded from this study. All participants provided written informed consent. The study protocol was reviewed and approved by the ethics committee of the Hokkaido University of Science and the Teine Keijinkai Hospital.

Transition milk was collected manually by a skilled nurse, on day 4 after delivery for women with vaginal delivery, and on day 5 of delivery for women with cesarean delivery. Mature milk was collected by the nurse manually on day 30 of postpartum period in the hospital. All transition milk and mature milk samples were collected immediately before breastfeeding at any time in midmorning on the day of sampling. During collection, the nurses wore gloves treated with an RNase inactivation agent (RNase Away; Molecular BioProducts, Inc., CA, USA). The breast milk samples (5–10 mL) were stored in 50 mL RNase-free centrifuge tubes, immediately transported to our laboratory using an ice box, and stored at − 80 °C in RNase-free microtubes until further analysis.

On the 30th day after delivery, the participants were administered a questionnaire to assess their milk-feeding practice and the use of medications and supplements during lactation. The participants who engaged in infant formula feeding or received medications (including selective serotonin reuptake inhibitors and adrenergic β_2_ stimulants, or supplements, including tryptophan, which have been reported to potentially influence milk production [25–27]) were excluded from the analysis.

### Infant formula sampling

Commercially available infant formulae manufactured by six Japanese companies, namely, Glico Co., Ltd., Bean Stark Snow Co., Ltd., Megmilk Snow Bland Co., Ltd., Morinaga Milk Industry Co., Ltd., Asahi Group Foods, Ltd., and Meiji Co., Ltd., were analyzed. These infant formulae were intended for consumption by infants aged less than a year and prepared according to the manufacturer’s instructions.

### Analysis of miRNA expression

The total RNA was extracted from breast milk and infant formula using a miRNAeasy serum/plasma kit (Qiagen, Hilden, Germany) [28] according to the manufacturer’s protocol. Before extraction, a fixed quantity of *Caenorhabditis elegans* miR-39-3p (cel-miR-39, Catalog No. 219610, Qiagen) as a spike-in control was added to 100 μL of breast milk or infant formula. The concentration and purity of the extracted total RNA were measured using a Nanodrop 2000 spectrometer (Thermo Fisher Scientific, MA, USA) and Agilent Bioanalyzer 2100 with RNA 6000 Nano Chips (Agilent Technologies, CA, USA) [29]. cDNA was synthesized using a TaqMan miRNA Reverse Transcription Kit (Thermo Fisher Scientific) [30]. miRNA levels were evaluated using the 7500 Fast Real-Time PCR System with TaqMan Universal Master Mix ΙΙ (Thermo Fisher Scientific). Commercially available pre-designed primer and probe sets were used for assessing miR-148a (Thermo Fisher Scientific, 000470), miR-125b (Thermo Fisher Scientific, 000449), and cel-miR-39 (Thermo Fisher Scientific, 000200). Quantitative values were obtained from the threshold cycle number. The miRNA levels were normalized to cel-miR-39 level and presented as the average relative transcript levels. All samples were analyzed in triplicate.

### Statistical analysis

The difference in mean levels of miR-148a and miR-125b between transition milk and mature milk were compared using the paired *t*-test. The difference in the expression of miR-148a and miR-125b in transition milk and mature milk between women with vaginal delivery and women with cesarean delivery was compared using the two-way analysis of variance (ANOVA) followed by Tukey–Kramer test. Comparison of miR-148a and miR-125b levels between mature milk and Japanese infant formulae were performed using one-way ANOVA followed by the Dunnett’s multiple test. Statistical analyses were performed using SPSS version 25.0 (IBM Corp., Armonk, NY, USA). Data are presented as mean ± standard deviation. Differences were considered statistically significant if their *p* - value was < 0.05. Sample size was constrained by available resources; we were able to recruit 16 women and analyze 22 samples within available resources.

## Results

### Patient characteristics

In this study, we aimed to investigate the changes in miR-148a and miR-125b levels during lactation and compare them with those in six infant formulae commercially available in Japan.

**Fig. 1:**
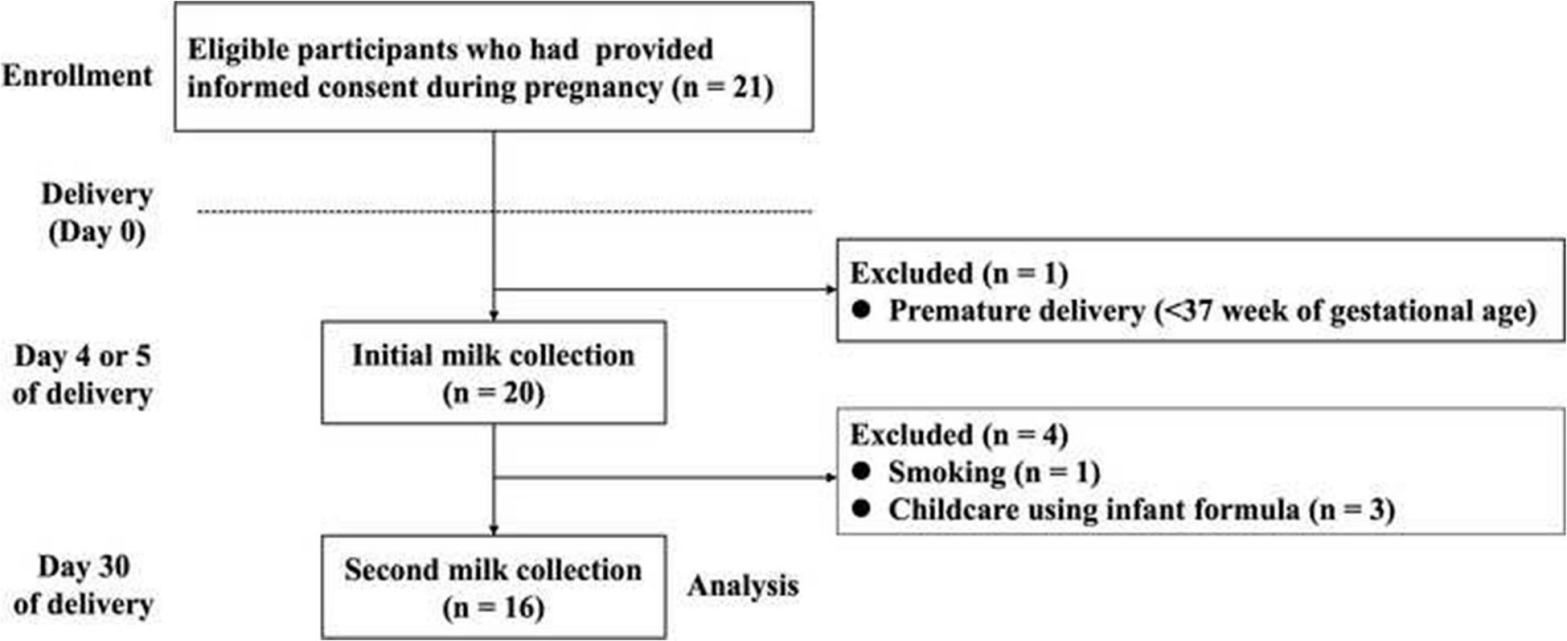
Flow diagram representing participants of this study

Among the 21 healthy Japanese lactating women initially included in the present study (Fig. 1), one underwent premature delivery (< 37 week of gestational age), one was a smoker, and three mainly used infant formula for baby feeding. Therefore, these five mothers were excluded from the analysis (Fig. 1). None of the participants had taken any medication or supplement. Finally, 16 lactating women were included in the study. Participant characteristics are summarized in Table 1.

**Table 1:** Characteristics of the 16 healthy Japanese lactating women included in the study

	Mean ± SD
Age (years)	30.72 ± 3.92 (21–37)
Gestational age (weeks)	39.52 ± 1.08 (38–41)
Delivery method	
Vaginal	9
Cesarean	7
Number of birth experience	
Primiparous	8
Multiparous	8
Body Mass Index (BMI) (kg/m^2^)	20.9 ± 2.28 (17.7–24.5)
Birthweight of infants (g)	3097.95 ± 361.71 (2702–3995)

Data are presented as mean ± standard deviation (SD). The value range is indicated in parentheses

### Comparison of miR-148a and miR-125b expression between transition and mature milk

First, we compared the expression levels of miR-148a and miR-125b between transition milk and mature milk. The mean relative expression of miR-148a in mature milk was significantly lower than that in transition milk (Table 2). The mean relative expression of miR-125b in mature milk was significantly lower than that in the transition milk (Table 2).

**Table 2:** Changes in hsa-miR-148a-3p and hsa-miR-125b-5p expression between transition and mature breast milk

Subject No.	hsa-miR-148a-3p		hsa-miR-125b-5p	
	Transition Milk	Mature Milk	Transition Milk	Mature Milk
1	1.99	0.45	2.32	0.93
2	1.51	0.49	0.58	0.26
3	0.78	0.34	0.66	0.50
4	1.04	0.62	0.54	0.31
5	1.07	0.72	1.15	0.36
6	0.59	0.37	0.86	0.52
7	0.33	0.17	0.43	0.19
8	0.64	0.09	1.04	0.23
9	0.12	0.06	0.23	0.15
10	0.06	0.26	1.88	0.44
11	0.56	0.22	0.83	0.15
12	1.09	0.06	1.33	0.05
13	0.79	0.16	0.57	0.15
14	1.62	1.01	1.39	0.60
15	1.82	1.01	1.16	0.56
16	1.21	0.61	1.02	0.70
Mean ± SD	1.00 ± 0.53	0.41 ± 0.31*	1.00 ± 0.53	0.38 ± 0.23*
*t* value	6.13		5.62	
Degree of freedom	15		15	

The level of hsa-miR-148a-3p and hsa-miR-125b-5p expression was analyzed using quantitative RT-PCR and normalized to that of the spike-in control cel-miR-39. The mean levels of hsa-miR-148a-3p or hsa-miR-125b-5p between transition milk and mature milk were compared using paired *t*-test (*P < 0.05). Data are presented as mean ± standard deviation (SD)

An in vivo study of goat milk showed that the relative expression of chi-miR-148a-3p in milk of early lactation was significantly higher than that of late-lactation [31]. In contrast, Chen et al. showed that bta-miR-148a expression in cow milk at one month of lactation tended to be higher than that at day 7 of lactation [32]. Some studies showed that the composition of milk, including the levels of proteins, fat, and lactose, varies during lactation to satisfy the age-dependent needs of infants [23, 24, 33, 34]. Although the mechanism underlying the decreased levels of miR-148a and miR-125b in the postpartum period remains unknown, we speculated that these changes are beneficial to infants.

Here, we found that the levels of miR-148a and miR-125b in transition milk obtained from some women (miR-148a: participant nos. 7 and 8, miR-125b: no. 9) were lower than the averages of miR-148a and miR-125b levels in mature milk, respectively. It is well known that stress exposure during lactation affects milk constituents and milk yield. Some studies have showed that stress decreases the yield of milk and concentrations of milk proteins such as casein in bovine and mice [27, 35, 36]. Additionally, the level of secretary immunoglobulin IgA in breast milk is negatively corelated with a negative Profile of Mood State, which is an index of psychological states such as tension-anxiety, depression-dejection, anger-hostility, fatigue, and confusion [37]. We could not elucidate the reasons for the phenomenon. Further studies should be performed to evaluate the changes in the miR-148a and mi-125b levels during lactation, including assessment of stress and living environment of lactating women.

### Comparison of miR-148a and miR-125b expression between women with vaginal and women with cesarean delivery

Second, we compared the levels of miR-148a and miR-125b between women with vaginal and women with cesarean delivery. The mean level of miR-148a was significantly lower in mature milk than in transition milk obtained from women with vaginal delivery (Table 3). Additionally, the mean level of miR-148a in mature milk obtained from women with cesarean delivery was significantly lower than that in transition milk obtained from women with vaginal delivery or women with cesarean delivery (Table 3).

**Table 3:**
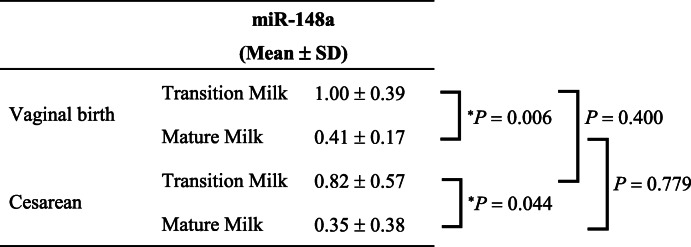
Comparison of hsa-miR-148a-3p levels in breast milk between women with vaginal or cesarean delivery.

The expression of hsa-miR-148a-3p was analyzed using quantitative RT-PCR and normalized to that of spike-in control cel-miR-39. The mean levels of hsa-miR-148a-3p among each breast milk sample obtained from women with vaginal delivery or women with cesarean delivery were compared using the two-way analysis of variance (ANOVA) followed by Tukey-Kramer test (^*^P < 0.05). Data are presented as mean ± standard deviation (SD).

The mean level of miR-125b was significantly lower in mature milk than in transition milk obtained from women with vaginal delivery (Table 4). Additionally, the mean level of miR-125b in mature milk obtained from women with cesarean delivery was significantly lower than that in transition milk obtained from women with vaginal delivery or women with cesarean delivery (Table 4).

**Table 4:**
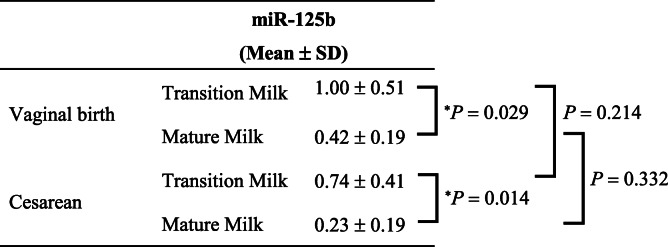
Comparison of hsa-miR-125b-5p levels in breast milk between women with vaginal or cesarean delivery.

The expression of hsa-miR-125b-5p was analyzed using quantitative RT-PCR and normalized to that of spike-in control cel-miR-39. The mean levels of hsa-miR-125b-5p among each breast milk sample obtained from women with vaginal delivery or women with cesarean delivery were compared using the two-way analysis (ANOVA) followed by Tukey-Kramer test (^*^P < 0.05). Data are presented as mean ± standard deviation (SD).

Furthermore, the levels of miR-148a and miR-125b in transition milk obtained from women with vaginal delivery tended to be higher than those in transition milk from women with cesarean delivery, respectively (Table 3 and 4). Ti et al. reported that the concentrations of sodium and potassium in breast milk obtained from women with cesarean delivery at 72–165 h (approximately days 3 to 7) after delivery were significantly lower than that in breast milk from women with vaginal delivery [38]. In the future, larger studies should be conducted to investigate the influence of cesarean delivery on miR148a and miR-125b expression in breast milk.

### Comparison of miR-148a and miR-125b expression between primiparous and multiparous women

Third, we compared the levels of miR-148a and miR-125b between primiparous and multiparous women. The mean level of miR-148a was significantly lower in mature milk than in transition milk obtained from primiparous women (Table 5). Additionally, the mean level of miR-148a in mature milk obtained from multiparous women was significantly lower than that in transition milk obtained from primiparous women or multiparous women (Table 5).

**Table 5:**
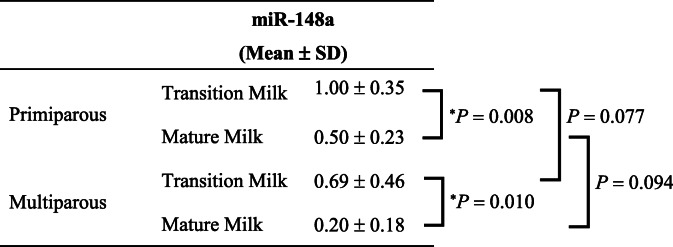
Comparison of hsa-miR-148a-3p expression in breast milk between primiparous and multiparous women.

The relative expression of hsa-miR-148a-3p was analyzed using quantitative RT-PCR and normalized to that of spike-in control cel-miR-39. The mean levels of hsa-miR-148a-3p among each breast milk sample obtained from primiparous or multiparous women were compared using the two-way analysis of variance (ANOVA) followed by Tukey-Kramer test (^*^P < 0.05). Data are presented as mean ± standard deviation (SD).

The mean level of miR-125b was significantly lower in mature milk than in transition milk obtained from primiparous women (Table 6). Additionally, the mean level of miR-125b in mature milk obtained from multiparous women was significantly lower than that in transition milk obtained from primiparous women or multiparous women (Table 6).

**Table 6:**
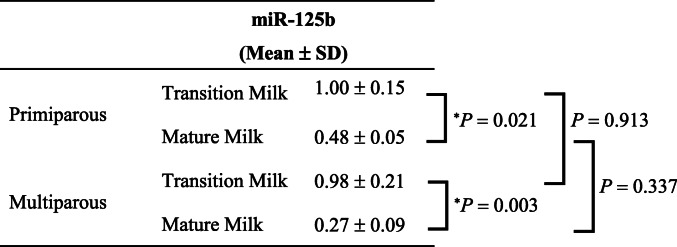
Comparison of hsa-miR-125b-5p expression in breast milk between primiparous and multiparous women.

The relative expression of hsa-miR-125b-5p was analyzed using quantitative RT-PCR and normalized to that of spike-in control cel-miR-39. The mean levels of hsa-miR-125b-5p among each breast milk sample obtained from primiparous or multiparous women were compared using the two-way analysis of variance (ANOVA) followed by Tukey-Kramer test and student’s *t*-test (^*^P < 0.05). Data are presented as mean ± standard deviation (SD).

Furthermore, the levels of miR-148a and miR-125b in transition milk obtained from primiparous women tended to be higher than those in transition milk from multiparous women (Table 5 and 6). Burianova et al. reported that primiparous women had a higher level of milk proteins in colostrum than multiparous women [39]. Lang et al. showed that primiparous women had a significantly higher density of mammary alveolus during early lactation than multiparous women [40]. In our study, slightly higher levels of miR-148a and miR-125b in breast milk obtained from primiparous women might be associated with a higher density of mammary alveolus.

In this study, we could not elucidate why the levels of miR-148a and miR-125b in transition milk were significantly higher than those in mature milk. During pregnancy, mammary epithelium differentiates and develops to gain milk production function via the stimulation of numerous endocrine hormones, such as estrogen, progesterone, and prolactin [41]. Additionally, the establishment and maintenance of tight junctions (TJs) in mammary epithelial cells are essential for lactation, and the functional integrity of TJs is established shortly after parturition [42]. Differentiated mammary epithelial cells during lactation regulates the transfer of blood constituents from circulating blood to milk [42]. These reports show that miR-148a and miR-125b transferred from the blood might remain in transition milk during pregnancy, and this may be a reason for the higher levels of miR-148a an miR-125b in transition milk than in mature milk.

A previous study reported that treatment with lactogenic hormones (a mixture with dexamethasone, insulin, and prolactin) increased bta-miR-148a-3p expression in bovine mammary epithelial cells [43]. This report implies that differentiated mammary epithelial cells may have a higher capacity of miR-148a production due to the stimulation of lactogenic hormones than undifferentiated cells. However, factors stimulating miR-125b expression in mammary epithelial cells have not been identified. Further studies are required to identify stimulating factors involved in miR-125b expression using human mammary epithelial cells.

### Comparison of miR-148a and miR-125b expression between breast milk and infant formulae

Finally, in the six infant formulae analyzed in the present study, miR-148a expression was less than 1/500th of that in mature breast milk (Table 7). Meanwhile, the level of miR-125b in all infant formulae was less than 1/100th of that in mature breast milk (Table 7).

**Table 7:** Comparison of hsa-miR-148a-3p and hsa-miR-125b-5p expression between mature breast milk and commercial infant formulae

		Mature milk (***n*** = 16)	Japanese Infant Formulae					
			a	b	c	d	e	f
miR-148a	Mean	1.00	5.70E-04*	1.38E-03*	6.34E-03*	1.41E-03*	6.60E-05*	3.39E-05*
	SD	0.76	5.32E-05	1.09E-04	6.00E-05	5.51E-04	3.01E-06	3.52E-05
miR-125b	Mean	1.00	3.06E-03*	8.24E-03*	3.78E-03*	6.87E-03*	3.93E-04*	2.02E-03*
	SD	0.64	7.79E-04	6.48E-04	3.57E-04	2.17E-03	1.80E-05	2.10E-04

The expression of hsa-miR-148a-3p (miR-148a) and hsa-miR-125b-5p (miR-125b) was analyzed using quantitative RT-PCR and normalized to that of spike-in control cel-miR-39. Six infant formulae (a–f) were analyzed. The levels of miR-148a and miR-125b in each infant formulae were compared with breast milk samples obtained from 16 Japanese healthy nursing women 30 days after delivery (mature milk). *P < 0.05, compared with mature milk, the Dunnett’s multiple test. Data are presented as mean and standard deviation (SD)

Infant formulae provide essential nutrition to infants who cannot consume breast milk. The formulae have been continuously improved to closely mimic the nutritional composition of breast milk [44]. In the present study, we focused on miR-148a and miR-125b, which are abundantly found in breast milk, and compared their expression in breast milk and infant formulae. In all six infant formulae, they were detected only in low quantities. Their sequences have high homology between humans and bovines [11], implying that the detected miRNAs in infant formulae may be derived from bovine miRNAs.

Further studies should explore whether miRNAs present in small amounts influence infant growth; specifically, the effects of several milk-derived miRNAs on infant development should also be investigated. Furthermore, the miRNA profiles of breast milk and infant formulae should be evaluated and compared. This information will be beneficial to the development of more nutritious infant formulae.

### Limitations

The present study had some limitations. Since it was a small-scale investigation conducted in Japanese lactating women from a single center, our observations should be regarded as a pilot study. The changes in miR-148a and miR-125b expression during lactation were investigated for only 30 days of the postpartum period. Additionally, self-reported information on breastfeeding implementation status and the use of medication and supplements over 30 days of the postpartum period might have certain limitations/bias. Furthermore, we did not evaluate the difference in the levels of miR-148a and miR-125b between days 4 and 5 after delivery in this study. Future studies with higher number of participants focusing on stress, situation of breastfeeding, and use of medication are required to understand the changes in miR-148a and miR-125b throughout lactation, and to confirm whether similar changes occur in multiethnic subjects.

## Conclusions

The levels of both miR-148a and miR-125b in human breast milk decreased after 30 days postpartum compared with those in the transition milk. This observation is novel regarding the physiological changes in milk components in humans. Further studies are required to investigate the reason for the observed reduction. Additionally, the expression of these miRNAs in infant formulae commercially available in Japan was found to be very low. This information emphasizes the importance of breastfeeding and can be beneficial for the development of more nutritious infant formulations.

## Data Availability

The datasets generated and/or analyzed during the current study are not publicly available due to privacy protection and ethical obligations but are available (in deidentified form) from the corresponding author on reasonable request.

## Abbreviations

ANOVA: Analysis of variance
BMI: Body Mass Index
DNMT1: DNA methyltransferase 1
MicroRNAs: miR-148a, hsa-miR-148a-3p; miR-125b, hsa-miR-125b-5p
TJs: Tight junctions
